# An exploration of the rapid transformation method for *Dunaliella salina* system

**DOI:** 10.1186/s13568-019-0905-3

**Published:** 2019-11-09

**Authors:** Guannan Song, Wan Wang, Lina Hu, Yu Liu, Aifang Li, Jingxia Du, Jiao Wang, Mengyuan Jia, Shuying Feng

**Affiliations:** 1 Medical College, Henan University of Science and Technology, No. 263 Kaiyuan Avenue, Luoyang, 471023 Henan China; 2 Medical Research Center, Henan University of Science and Technology, Luoyang, 471023 Henan China

**Keywords:** *Dunaliella salina*, Transformation, Salt gradient, High efficiency, Rapid method

## Abstract

As a new expression system, *Dunaliella salina* (*D. salina*) has bright prospects and applications in various fields. However, its application is currently restricted because of the low expression and instability of foreign gene in *D. salina* cells. During genetic operation, transformation is a crucial step for genes expression in *D. salina* system. Although several transformation methods are existing currently, many inherent deficiencies and limitations still can be found in actual practice. Thus, we attempted to set up a rapid transformation method using the change of salt concentrations for *D. salina*. Based on osmotic pressure difference, exogenous genes can be spontaneously transferred into *D. salina* cells. After that, transformed *D. salina* cells were subjected to histochemical and molecular analysis. The results showed that the reporter gene, beta-glucuronidase genes were successfully expressed in the positive transformants, and detected in all of transformed cells by PCR analysis. Moreover, different transformation parameters, containing the salt gradient, time, dye dosage and Triton X-100 concentration, were optimized to obtain an optimal transformation result. Taken together, we preliminarily established a rapid transformation method with the features of fast, simple, economic, and high-efficient. This method will provide a strong genetic manipulation tool for the future transformation of *D. salina* system.

## Introduction

Currently, microalga as a versatile expression system that has been widely used in the fields of vaccine (Dehghani et al. 2018), bio-based chemicals (Ng et al. 2017), metabolic engineering (Anila et al. 2016), pharmaceutical engineering (Zhang et al. 2018), and so on. Among them, since *Dunaliella salina* (*D. salina*) offers numerous special advantages, it has been exploited as a novel expression system for production of recombinant proteins (Poungpair et al. 2014; Feng et al. 2014a, b, c). Although several exogenous genes have been transformed into *D. salina*, such as the human canstatin (Feng et al. 2014a), soybean kunitz trypsin inhibitor (Chai et al. 2012), and white spot syndrome virus VP28 (Feng et al. 2014b), and interferon-thymosin fusion proteins (Zhang et al. 2018), most of them exhibited the low and instable expression in nuclear system of *D. salina*. Thus, improving the expression level of foreign gene in *D. salina* cells is an urgent issue for current study.

In the genetic operation of *D. salina*, transformation is a key step for expression of exogenous genes. So far, several transformation methods have been established for *D. salina* system, like electroporation (Lü et al. 2009), bombardment particle (Tan et al. 2005), and glass beads (Feng et al. 2009). Each of them has some inherent disadvantages with respect to simplicity, economy, operability and reproducibility, which made them have great limitations in the practical transformation of *D. salina*. Given this, more efficient and rapid approach should be established for future transformation of *D. salina.*

Due to it can grow in 0.1–5 M salt concentration, *D. salina* has the remarkable halotolerant ability in practical culture (Feng et al. 2014b). Based on this feature, we attempted to establish a rapid transformation method using the difference of salt gradients. When salt gradient decreased from high to low, cells permeability was increased instantaneously and then exogenous plasmids introduced simultaneously into *D. salina* cells. Moreover, to obtain an optimal transformation rate, we optimized the different transformation parameters which including salt gradient, time, dye dosage and Triton X-100 concentration. Under the optical transformation conditions, exogenous genes could be high-efficiently transferred into *D. salina* cells. Our study showed that a simple and rapid transformation method successfully established for transformation of *D. salina* cells.

## Materials and methods

### Algal strain and culture condition

The *D. salina* strain UTEX-1644 was purchased from the Culture Collection of Algae (University of Texas, USA). Under light intensity of 50 µM photon m^−2^s^−1^ with a 12 h-light/day, *D. salina* cells were cultured in 2 L beaker with the modified PKS medium at 26 °C (Feng et al. 2009). The modified liquid medium consist of 1.5 M NaCl, 1 µM CuCl_2_·2H_2_O, 10 mM KNO_3_, 50 mM NaHCO_3_, 0.4 mM KH_2_PO_4_, 185 µM H_3_Bo_3_, 2 µM FeCl_3_·6H_2_O, 5 mM MgSO_4_·7H_2_O, 5 µM EDTA, 1 µM (NH_4_) Mo_7_O_24_·4H_2_O, 7 µM MnCl_2_·4H_2_O, 1 µM ZnCl_2_, 1 µM CoCl_2_·6H_2_O, and 0.2 mM CaCl_2_. When growth reached logarithmic phase (about 10^5^–10^6^ cells/mL), *D. salina* cells were harvested by centrifugation for future use. After washed three times with the fresh medium, *D. salina* cells were re-suspended and the cells concentration was adjusted to about 10^6^ cells/mL for further use.

### Manipulation protocols with the salt gradients

Ethidium bromide (EB) was purchased from Solarbio Science & Technology Co. Ltd (Beijing, China); and was formulated into different concentration gradients. Triton X-100 was purchased from Baoxin biotechnological Co. Ltd (Luoyang, China), and was made as a 0.1% stock solution. After 8 h cultured in 1 M medium, *D. salina* cells were harvested and then re-suspended with the 0.1 M fresh medium. Meanwhile, different concentrations of Triton X-100 (5, 10, 15, 20, 25 and 30 µL) and EB (1.25%, 2.5%, 3.75%, 5%, 6.25% and 7.5%) were immediately added to 1 mL *D. salina* separately. And then, this mixture was blended briefly using inverting tube. *D. salina* cells were observed quickly at the point in time of 60 s, 90 s, 120 s, 150 s and 180 s under a fluorescence microscope. In this experiment, four factors were determined orderly which including salt gradient, time, TritonX-100 concentration and EB amount. When one variable of four variables was optimized, the other three parameters were unchanged as described above. The independent experiments were repeated three times at least.

### Transformation of *D. salina* cells with the plasmids

**Fig. 1:**
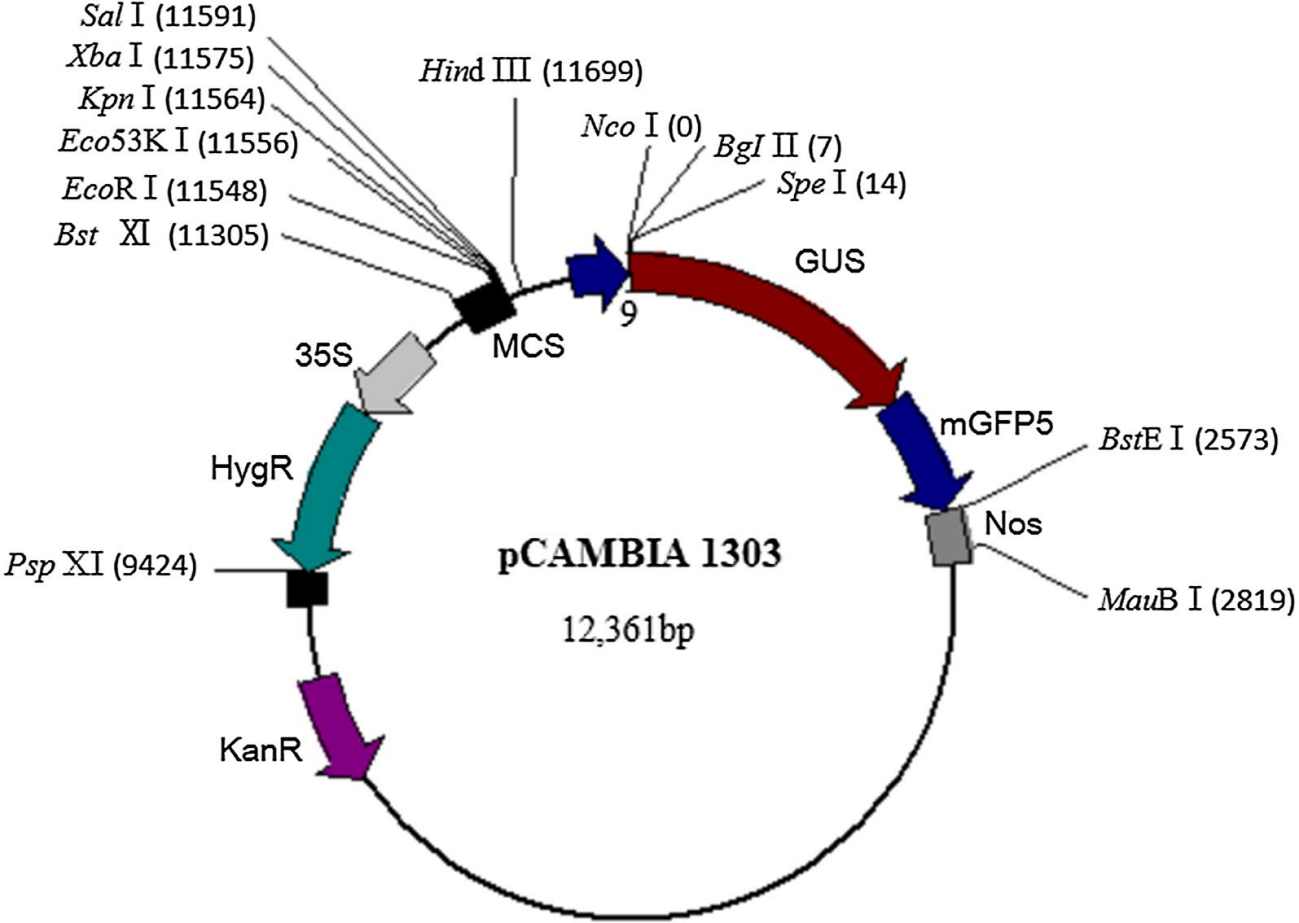
*35S* CaMV 35S promoter, *GUS* β-glucuronidase, *mGFP5* modified green fluorescent protein, *Nos* nos terminator. Restriction endonuclease sites are indicated

The plasmids pCAMBIA1303 were purchased from Guangyu biotechnological Co. Ltd (Shanghai, China), which including a selective gene (Kan^+^) and a report gene (β-glucuronidase, GUS). The modified plasmids pCAMBIA1303-N, in which GUS gene fragment was deleted, were constructed in our lab that used as the negative control plasmids. The restriction endonuclease sites and other features were indicated in Fig. 1. Under the optimal transformation conditions, *D. salina* cells were transformed with the plasmids pCAMBIA1303 and pCAMBIA1303-N respectively. In the 1 mL culture of *D. salina* cells, 5.46 µg vector DNA was added and then mixed briefly at 800 *g* on a Vortex mixer for 60 s. More than 90% of surviving transformed *D. salina* cells were cultured in liquid medium at 26 °C with a 12 h-light/day for 48 h. After that, *D. salina* cells were collected and then analyzed with the histochemical staining (Feng et al. 2009) and PCR analysis (Chen et al. 2001). The negative control and blank control were treated with the same processes using the negative control plasmids and the blank control (liquid medium), respectively. The repeated experiments were conducted three times at least.

### Statistical analysis

In this study, all the numbers came from independently repeated experiments at least three times. The transformants were counted by the number of blue cells in total transformed cells. Using SPSS version 17.0, all data were carried out with one-way analysis of variance. In which, the value *P* < 0.05 was considered statistically significant in all statistical analyses.

## Results

### Morphology of stained *D. salina* cells with EB

**Fig. 2:**
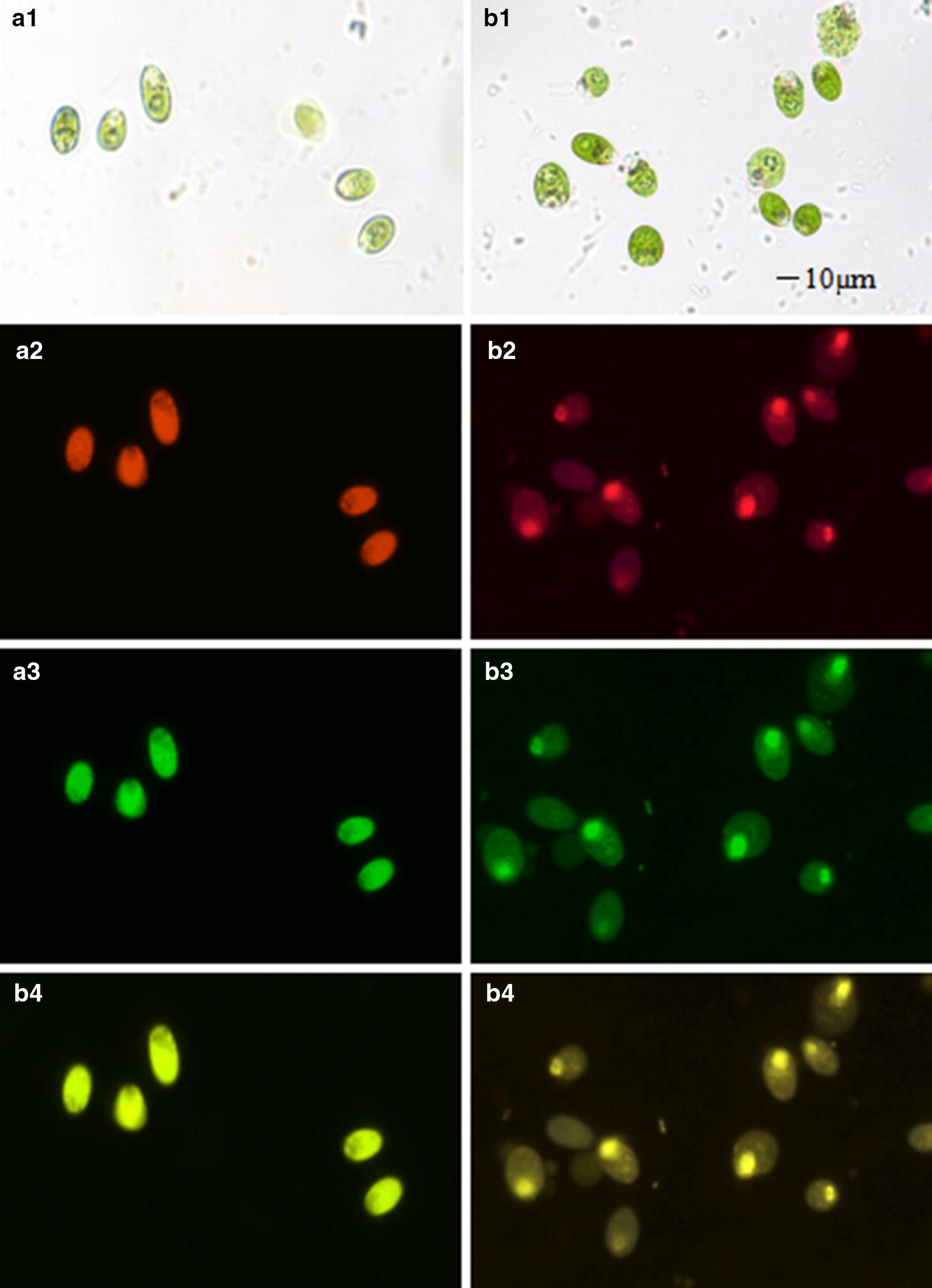
**a1**–**a4** Represented the wild type *D. salina* cells under the light, red fluorescence (excitation wavelength 530–550 nm, emission wavelength 575–635 nm), green fluorescence (excitation wavelength 460–490 nm, emission wavelength 510–560 nm), and the merged fluorescence (red with green), respectively; **b1**–**b4** represented the stained *D. salina* cells under the corresponding mentioned above light and fluorescence sources

To measure the difference of treated *D. salina* lines, cells with fluorescence were counted under the fluorescence microscopy. After EB staining, morphology and color of each cell line was observed under different light sources (Fig. 2a1, b1). Under the red and green fluorescent sources, wild *D. salina* cells shown the red and green color correspondingly (Fig. 2a2, a3). Meanwhile, stained *D. salina* cells appeared the lighter color under the same fluorescent source (Fig. 2b2, b3). But the cells nucleus shown a deeper color under the red and green fluorescent sources. Under the merged fluorescence sources (red with green), wild type and stained cells shown the lighter green color and yellow color separately (Fig. 2a4, b4). After statistical analysis, the number of cells with fluorescence could approach up to 100%.

### Optimization of different parameters

**Fig. 3:**
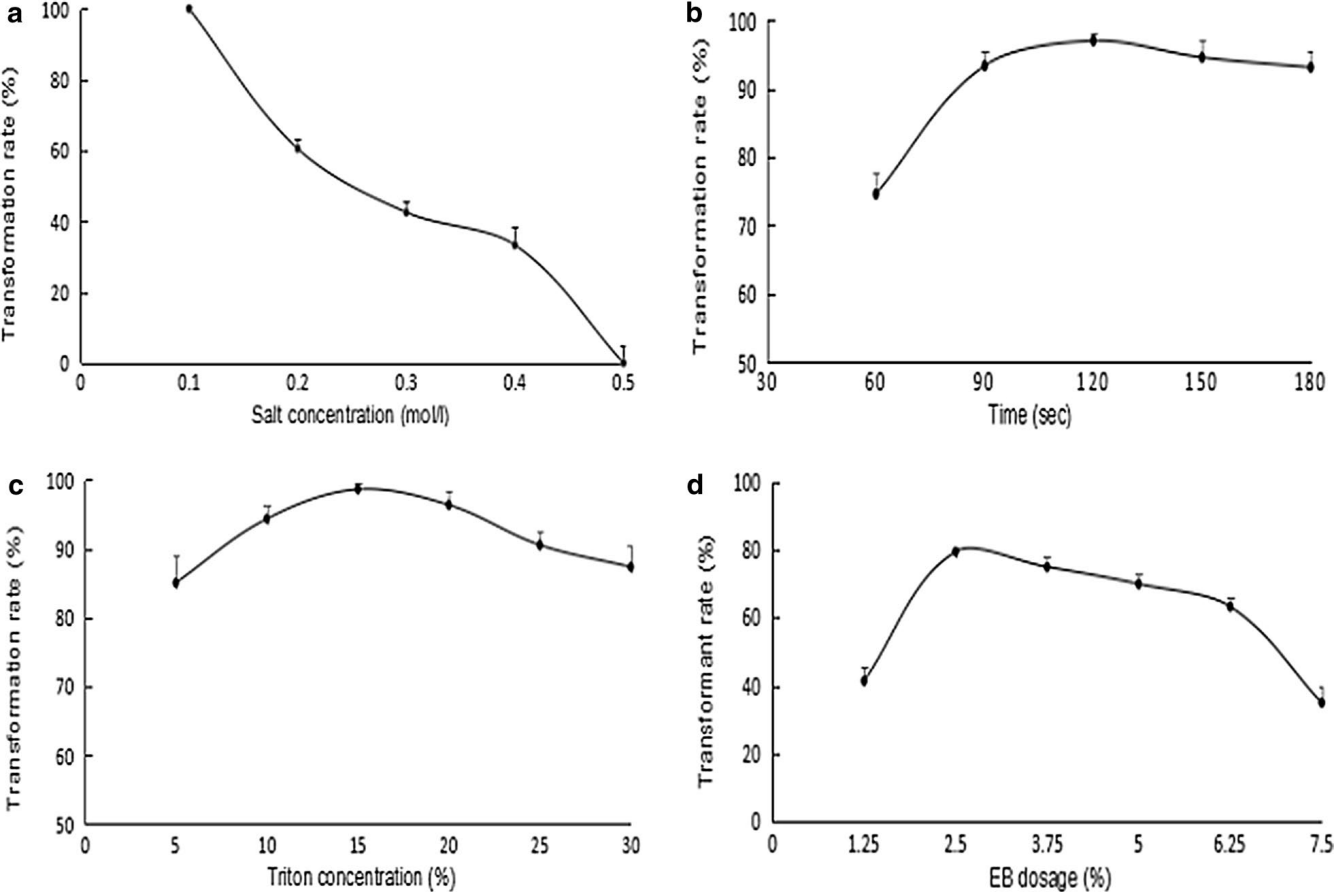
**a**–**d** Denoted the effects of salt gradient, time, concentration of Triton X-100 and EB on the number of transformants, respectively. **a** The number of transformants increased significantly along with the decrease of salt concentration. When the salt concentration was less than 0.1 M, the number of transformants decreased significantly with the highest value. **b** The number of transformants increased noticeably with the extension of transformation time. Following the further extension of time, the number of transformants did not increase correspondingly. **c** The increasing of transformants was associated with the Triton X-100 concentration in a dose-dependent manner. When concentration of Triton X-100 was over than 3.75%, the number of transformants was significantly decreased because of the strong dissolution. **d** The maximum number of transformants can be achieved with the 10% EB concentration, and decreased significantly at the other concentrations

To obtain the optimal transformation rate, four parameters have been optimized in turn which containing salt gradient, time, EB and TritonX-100 concentration in this study. Firstly, transformants increased most significantly along with the salt concentration decreasing (Fig. 3a). When salt concentration decreased to 0.1 M, *D. salina* cells with fluorescence reached as high as 100% (*P* < 0.01). However, if salt concentration was less than 0.1 M, the number of transformants decreased distinctly. Secondly, transformation time has a great influence on the transformation results. Between 60 and 150 s, the number of transformants increased noticeably with the extension of time, and the optimal time was from 90 to 120 s (Fig. 3b, *P* < 0.05). Followed further extension of time, positive transformants did not increase correspondingly due to cells rupture. Thirdly, the number of transformants was closely associated with the Triton X-100 concentration. As shown in Fig. 3c, transformants were increased with the Triton X-100 concentration in a dose-dependent manner. Within range of 5–15 µL, the number of transformants increased from 85 to 98.6%. When TritonX-100 concentration was higher than 15 µL, a significant reduction of transformants was observed due to the strong dissolution of Triton X-100 (Fig. 3c, *P* < 0.05). Finally, the maximum number of transformants was obtained with 3.75% EB concentration under mentioned above conditions (Fig. 3d, *P* < 0.05). But as the EB concentration increased, the number of transformations did not improve correspondingly. After completely analyzed the transformation results, the ideal transformation rate could be achieved under the following conditions: 10% EB and 15 µL 0.1% Triton X-100 were added to 1 mL culture with the treatment of 90–120 s in 0.1 M salt concentration.

### Detection of GUS gene expression

**Fig. 4:**
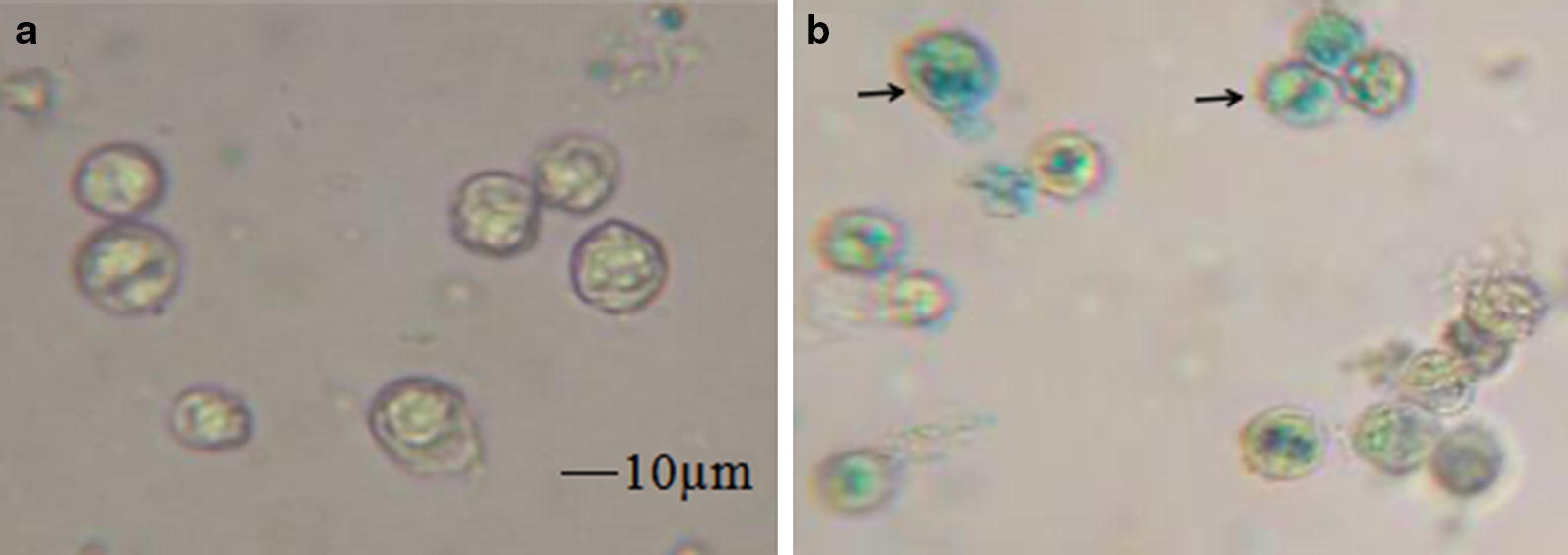
**a** Negative control: *D. salina* cells were transformed with the plasmids pCAMBIA1303-N; **b** transformed group: *D. salina* cells were transformed with the plasmids pCAMBIA1303. Arrow represented the positive transformants with the blue color

**Fig. 5:**
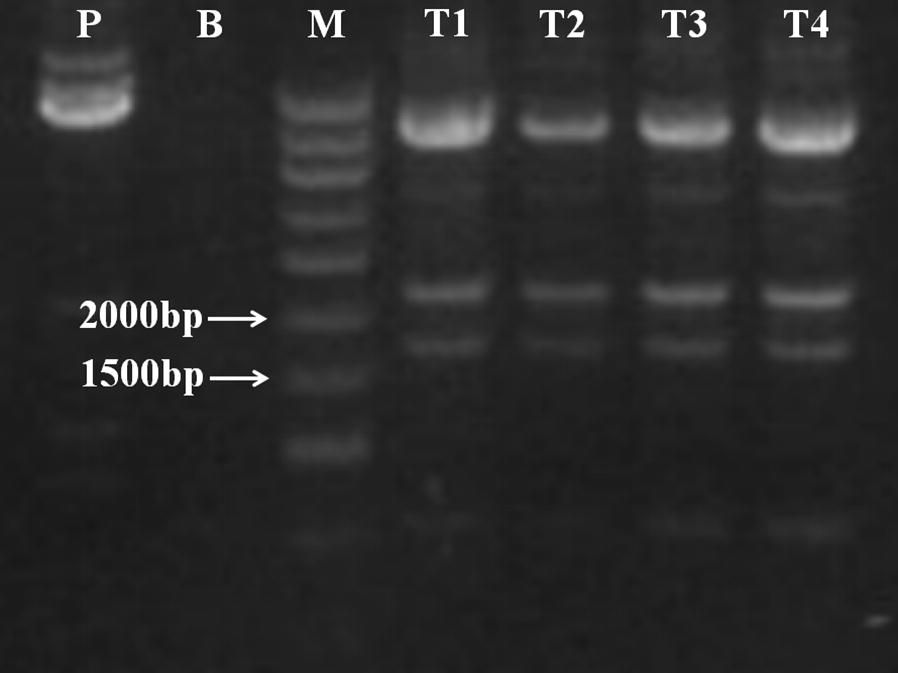
*P* plasmid pCAMBIA1303, *B* blank control, *M* DNA ladder marker, *T1–T4* different transformed *D. salina* cells lines

After histochemical staining, positive transformants showed the blue color partially (Fig. 4b), while negative control group was not shown (Fig. 4a). This transformation result was further confirmed by PCR analysis. As shown in Fig. 5, GUS-specific products of 1807 bp were detected in the all of transformed lines, but not in the negative control. The size of amplified fragments was consistent with the positive control. These results indicates that exogenous genes have been introduced into *D. salina* cells and further successfully expressed in the cells. The genetic stability analysis of transformed *D. salina* strains will be studied in next works.

## Discussion

To improve the gene expression in *D. salina* cells, and then facilitate the maturation of *D. salina* system, a robust transformation tool is essential for production of recombinant proteins. Since the shortcomings of current methods, we attempted to explore a simple and rapid transformation method using salt gradient for *D. salina*. Using EB as the staining dye, different transformation parameters were fast determined in the study. Among them, effect of salt concentration is the most obvious factor on the transformation rate. The transformation rate increased significantly along with the decrease of salt concentration. When salt concentration was less than 0.1 M, the transformation rate greatly reduced due to the water absorption of cells that ended up with the cells rupture. Although followed the increasing of time extension, the transformation rate declined distinctly owing to cells lysis when time was more than 120 s. Because Triton X-100 can dissolve the lipid bilayer of cells membrane, the tremendous destruction of Triton X-100 leads to a remarkable decrease of the number of transformants when its concentration was over than 15 µL. Collectively, in present study, the optimal transformation conditions was determined as follows: adding 15 µL 0.1% Triton X-100 and 10% EB to 1 mL culture (0.1 M salt concentration) with the treating time of 90–120 s.

In this study, relied on the features of quick, high sensitive, efficient staining, EB was used to rapidly identify the transformation results. Although the transformation of EB is different from that of plasmids, its transformation processes and parameters can be referred to the next transformation work of *D. salina* system. Here, we preliminarily stated the feasibility of this method for transformation of *D. salina* cells. Compared with the other methods, it has a significant transformation efficiency and the minimal damage to cells (Lü et al. 2009; Tan et al. 2005; Feng et al. 2009; Chai et al. 2012). However, the other transformation parameters were not optimized for transformation of plasmids into *D. salina* cells, which would be deeply studied in next works. The positive transformants were only identified at the level of cells and nucleic acid. The bio-activities of recombinant proteins were still not analyzed, which was the work in progress. Taken together, all the results demonstrated that an alternative rapid method has been successfully established for *D. salina* transformation. And it will provide a strong genetic manipulation tool for the future transformation of *D. salina* system.

## Data Availability

The data of this article is included within the article. And also, the data and materials can also be requested from the corresponding author and the first author.

## Abbreviations

*D. salina*: *Dunaliella salina*
EB: ethidium bromide
GUS: β-glucuronidase
